# Risk factors associated with mortality and pathogen characteristics of bloodstream infection-induced severe sepsis in the pediatric intensive care unit: a retrospective cohort study

**DOI:** 10.3389/fcimb.2025.1492208

**Published:** 2025-02-03

**Authors:** Jian Chen, Haixin Huang, Ruichen Zhang, Yueqiang Fu, Chunmei Jing

**Affiliations:** ^1^ Department of Critical Care Medicine, Children’s Hospital of Chongqing Medical University, National Clinical Research Center for Child Health and Disorders, Ministry of Education Key Laboratory of Child Development and Disorders, Chongqing Key Laboratory of Child Rare Diseases in Infection and Immunity, Chongqing, China; ^2^ Department of Pediatric Critical Care Medicine, Sichuan Provincial Women's and Children's Hospital, Chengdu, China; ^3^ Department of Clinical Laboratory, Children’s Hospital of Chongqing Medical University, National Clinical Research Center for Child Health and Disorders, Ministry of Education Key Laboratory of Child Development and Disorders. Chongqing Key Laboratory of Child Rare Diseases in Infection and Immunity, Chongqing, China

**Keywords:** bloodstream infection, sepsis, risk factors, intensive care units, children, mortality

## Abstract

**Background:**

Bloodstream infection (BSI)-induced severe sepsis is a common cause of mortality, frequently resulting in septic shock and multiple organ dysfunction syndrome (MODS). This study aimed to analyze mortality risk factors and summarize pathogen characteristics associated with BSI-induced severe sepsis in the pediatric intensive care unit (PICU).

**Methods:**

This retrospective study was conducted at a tertiary pediatric hospital between January 2015 and December 2023, encompassing children with BSI-induced severe sepsis in the PICU. Clinical characteristics, laboratory parameters, pathogen characteristics, and drug resistance profiles of the patients were collected. Clinical and laboratory indicators along with pathogen characteristics were summarized. Logistic regression analysis was employed to identify independent risk factors associated with 28-day mortality.

**Results:**

A total of 192 patients with bloodstream infection (BSI)-induced severe sepsis were identified, with a 28-day in-hospital mortality rate of 36.98% (71/192). The incidence of septic shock (42.1% *vs*. 69%, P < 0.001) and AKI (14% *vs*. 31%, P = 0.005) was significantly lower in the survival group compared to the non-survival group. In multivariate analysis, independent risk factors for 28-day mortality were the pediatric sequential organ failure assessment (pSOFA) score (OR 1.176; 95% CI: 1.046-1.321, *p* = 0.007) and the P/F value (OR 0.994; 95% CI: 0.991-0.997, *P <* 0.001). Double organism growth was detected in 8 cultures, and a total of 200 pathogenic bacteria were isolated from all blood cultures. Of these, 110 strains (55.0%) were Gram-negative bacteria, 88 strains (44.0%) were gram-positive bacteria, and 2 strains (1.0%) were *Candida albicans*. The most commonly isolated pathogens were *Staphylococcus aureus, Coagulase-negative Staphylococcus, and Escherichia coli*. The detection rate of carbapenem resistance (CR) in *Acinetobacter baumannii* (66.7%) was higher than that in *Pseudomonas aeruginosa* (15.4%). The detection rates of extended-spectrum cephalosporin resistance (ECR) and fluoroquinolone resistance (FQR) in *Escherichia coli* (*E. coli*) were higher than those in *Klebsiella pneumoniae*.

**Conclusion:**

In the PICU, higher mortality was observed in children with BSI-induced severe sepsis who presented with elevated pSOFA scores and low P/F values. *Acinetobacter baumannii* exhibited the highest levels of CR and FQR, while *Escherichia coli* demonstrated the highest level of ECR.

## 1 Introduction

Bloodstream infection (BSI) is defined as the presence of microorganisms in the bloodstream, as confirmed by a positive blood culture (BC) in a patient exhibiting clinical signs of infection (Laupland and Leal, 2020). Sepsis is a life-threatening condition resulting from a dysregulated immune response to infection that leads to organ dysfunction (Singer et al., 2016; Rhee et al., 2019). In 2020, the World Health Organization (WHO) reported that sepsis affects 49 million people and causes 11 million deaths globally each year, with many of the victims being children. Sepsis also disables millions more. The WHO recognized sepsis as a major public health issue and urged all United Nations (UN) member states to enhance sepsis prevention, recognition, and management (Singer et al., 2016; Rudd et al., 2020; World Health Organization, 2020). In recent years, the incidence of bloodstream infections (BSI) has been increasing, particularly among the young and elderly populations (Kontula et al., 2021). Approximately one out of three episodes of bacteremia are associated with organ dysfunction, according to a recent large population-based study. BSI-induced severe sepsis is a common cause of death and frequently results in septic shock and multiple organ dysfunction syndrome (MODS) (Weiss et al., 2020; Agyeman et al., 2017). BSI or septic shock remain an important cause of morbidity and mortality (Weiss et al., 2015; Martinón-Torres et al., 2018), with mortality rates reported as high as 40% (Weiss et al., 2015; Wang et al., 2014; Dou et al., 2024; Allel et al., 2023). Multiple analyses indicate that BSIs in the ICU significantly increase both the length of stay (LOS) and healthcare costs (Goudie et al., 2014; Timsit et al., 2020; Tabah et al., 2022). Bloodstream infection (BSI) presents a major challenge in the critically ill (Munro et al., 2024). Infection prevention and control efforts should prioritize preventing BSIs in the youngest age groups, particularly in neonatal and pediatric intensive care units (Zingg et al., 2017).

Bloodstream infections (BSI) can be caused by a variety of pathogens, and the microbiology of ICU BSIs varies globally (Munro et al., 2024). Understanding the clinical features and distribution of pathogenic bacteria in BSI-induced severe sepsis in local ICUs is crucial for treating severely infected children. In this study, we conducted a retrospective analysis to identify the risk factors for mortality and to characterize the pathogens associated with BSI-induced severe sepsis in the pediatric intensive care unit (PICU).

## 2 Material and methods

### 2.1 Study design

This retrospective study was conducted using clinical data of children with BSI−induced severe sepsis in the pediatric intensive care unit (PICU) at a tertiary pediatric hospital between January 2015 and December 2023. This study was approved by the Institutional Review Board of the hospital. Patient data were anonymized and de-identified prior to analysis. Due to the retrospective nature of the study, the requirement for informed *CoNS*ent was waived. The inclusion criteria were as follows: 1) patients aged 1 month to 18 years admitted to the PICU, 2) positive blood culture during hospitalization, and 3) meeting the diagnostic criteria for severe sepsis in children. The exclusion criteria were: 1) negative blood culture during hospitalization, and 2) isolated organisms identified as contaminants or fixed values.

### 2.2 Microbiologic methods

Blood samples were collected from peripheral veins of the central venous catheter. Each 1-5 mL blood sample was immediately inoculated into a culture bottle and transported to the laboratory. Blood cultures were processed BD BACTEC FX 400 automatic bacteria culture system. Drug susceptibility tests were performed using the conventional minimum inhibitory concentration (MIC) test with BD phoenix TM 100 automated microbiology system. This study was to examine the prevalence of four important drug-resistance phenotypes: difficult-to-treat resistance (DTR), fluoroquinolone resistance (FQR), carbapenem resistance (CR), and extended-spectrum cephalosporin resistance (ECR).

### 2.3 Data collection

We collected the laboratory indicators and scores within 24 hours of the first positive blood culture sample sent for testing. The following demographic and laboratory data were collected: age, body weight, gender, white blood cell (WBC), C-reactive protein (CRP), Procalcitoninalanine (PCT), albumin (ALB), and serum creatinine (SCr). The P/F value (PaO_2_/FiO_2_), pediatric sequential organ failure assessment (pSOFA) scores (Mohamed El-Mashad et al., 2020), International Society on Thrombosis and Hemostasis (ISTH) disseminated intravascular coagulation (DIC) scores, sepsis-induced coagulopathy (SIC) scores, underlying conditions, and Nosocomial infection were obtained. Comorbidities such as septic shock, respiratory failure, liver function impairment, and acute kidney injury (AKI) were also evaluated. Furthermore, data on the need for continuous renal replacement therapy (CRRT) and mechanical ventilation (MV), duration of CRRT and MV, as well as length of hospital stay and stay in the PICU were recorded.

### 2.4 Definitions

Sepsis: SIRS in the presence of or as a result of suspected or proven infection. Severe sepsis: sepsis plus one of the following: cardiovascular organ dysfunction or acute respiratory distress syndrome or two or more other organ dysfunctions. Septic shock: sepsis and cardiovascular organ dysfunction, cardiovascular dysfunction: despite administration of isotonic intravenous fluid bolus 40 mL/kg in 1 hr., decrease in BP (hypotension) 5th percentile for age or systolic BP 2 SD below normal for age, or need for vasoactive drug to maintain BP in normal range (dopamine 5 g/kg/min or dobutamine, epinephrine, or norepinephrine at any dose), or two of the following: 1. Unexplained metabolic acidosis: base deficit 5.0 mEq/L.2. Increased arterial lactate 2 times upper limit of normal; 3. Oliguria: urine output 0.5 mL/kg/hr. 4. Prolonged capillary refill: 5 secs. 5. Core to peripheral temperature gap 3°C (Goldstein et al., 2005). Nosocomial infection was defined as the first positive blood culture was drawn after day 2 of the hospital admission, with no primary diagnosis of infectious disease present on admission (as defined with International Classification of Disease (ICD) diagnosis codes and Major Diagnostic Categories). Respiratory failure is the inability of the respiratory system to maintain oxygenation or eliminate carbon dioxide, resulting in hypoxemia (PaO_2_ < 60 mmHg) with or without hypercapnia (PaCO_2_ > 50 mmHg), or the need for noninvasive or invasive ventilator support. Liver function impairment was defined as transaminase levels three times above the upper limit of normal. Acute kidney injury is defined as an increase in serum creatinine levels by at least 0.3 mg/dl within 48 hours or 1.5−fold the baseline, which is known or presumed to have occurred within the preceding 7 days, or-according to the urine output criterion-urine volume less than 0.5 ml/kg/hour for at least 6 hours. Contaminants: The most frequently identified contaminants in cultures were *Staphylococcus epidermidis* and *Staphylococcus hominis*. Critical value reports from blood cultures were systematically monitored, and clinicians assessed whether these organisms were true pathogens or contaminants based on the patient’s clinical presentation and relevant laboratory parameters. Difficult-to-treat resistance (DTR) was defined as resistance or intermediate resistance *in vitro* to all β-lactam categories, including carbapenems and fluoroquinolones. Carbapenem resistance (CR) was defined as resistance *in vitro* to imipenem or meropenem. Extended-spectrum cephalosporin resistance (ECR) was defined as resistance *in vitro* to ceftriaxone or cefepime (excluding natural drug resistance). Fluoroquinolone resistance (FQR) was defined as resistance *in vitro* to ciprofloxacin or levofloxacin.

### 2.5 Outcomes analysis

The primary outcome was 28-day in-hospital mortality, and the secondary outcome was the distribution of pathogens in bloodstream infection-induced severe sepsis.

### 2.6 Statistical analysis

Raw data was firstly processed by Whonet 5.6 software. Data processing was performed using Graphpad Prism 8.4.2 for Windows (GraphPad Software, San Diego, California, USA) and SPSS 26.0 software packages (IBM Corp., Armonk, NY, USA). We used the median (interquartile range or IQR) and proportion (percentage) to describe continuous and categorical data, respectively. The Manne-Whitney U test was used to compare two medians, and the chi-square test was used to compare proportions. Multiple logistic regression was employed to identify independent risk factors, controlling for confounding variables. Statistical significance was defined as P < 0.05 (two-tailed).

## 3 Result

### 3.1 Study population

**Figure 1:**
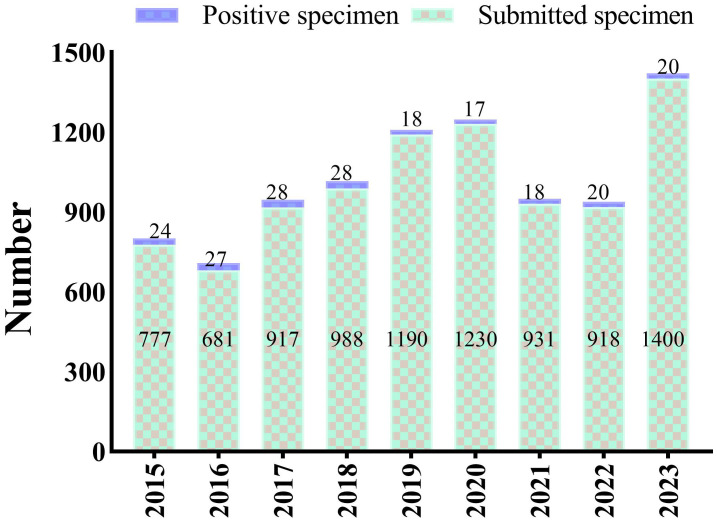
Annual number of blood cultures including the submitted specimens and positive specimens, from 2015 to 2023.

During the study period, a total of 9032 blood cultures were collected from patients suspected of bloodstream infections (BSI). Among these, 382 cultures tested positive. After excluding possible contaminants and duplicates, we analyzed 200 episodes confirmed as bloodstream infections leading to severe sepsis. The annual positive rates in the 9 years from 2015 to 2023 were 3.09%, 3.96%, 3.05%, 2.83%, 1.51%, 1.38%, 1.93%, 2.18%, and 1.43%, respectively (**Figure 1**).

### 3.2 Characteristics of patients diagnosed with BSI-induced severe sepsis

One hundred and ninety-two cases of bloodstream infection-induced severe sepsis were identified. The clinical characteristics of these patients are summarized in **Table 1**. We categorized the children into survival and non-survival groups, and their clinical characteristics are presented in **Table 1**. The 28-day in-hospital mortality rate was 36.98% (71/192).

**Table 1:** Clinical characteristics and outcomes associated with blood culture-proven bacterial sepsis in 192 children.

	All patients (n=192)	Survivors (n=121)	Non-survivors (n=71)	P value
Basic information				
Age,(month) (median, IQR)	16(6-84)	18(7-84)	11(6-96)	0.571
Weight, (kg) (median, IQR)	10.25(7-22.75)	11(7-23)	10(7-22)	0.735
Gender, (male) n (%)	114(59.4%)	73(60.3%)	41(57.7%)	0.726
Laboratory index				
WBC(×10^9^/L) (median, IQR)	8.41(4.59-15.19)	9.67(6.07-16.5)	5.96(1.55-12.47)	<0.001
CRP (mg/l) (median, IQR)	33(8-73)	30(8.93-73.5)	34(8-64)	0.747
PCT (ng/ml) (median, IQR)	9.21(1.38-55)	6.34(0.79-55)	14.54(2.60-98.44)	0.039
ALB (g/l) (median, IQR)	28.4(23.88-33.78)	30.4(25.5-36.3)	26.4(21.7-29.6)	<0.001
SCr (mmol/l) (median, IQR)	32.1(21.1-55.38)	28.2(18.2-47.75)	41(25-78.8)	<0.001
P/F value (median, IQR)	308.04(164.76-407.68)	345.71(259.57-431.95)	147.06(84.29-323.75)	<0.001
pSOFA (median, IQR)	9(6-12)	8(5-10)	12(8-14)	<0.001
ISTH-DIC (median, IQR)	3(3-5)	3(2-4)	5(3-6)	<0.001
SIC (median, IQR)	4(2-5)	3(2-4)	4(3-6)	<0.001
Clinical presentation				
Septic shock (n, %)	100(52.1%)	51(42.1%)	49(69%)	<0.001
Respiratory failure (n, %)	158(82.3%)	98(81%)	60(84.5%)	0.539
Liver function damage (n, %)	108(56.3%)	63(52.1%)	45(63.4%)	0.128
AKI (n, %)	39(20.3%)	17(14%)	22(31%)	0.005
*Underlying conditions (n, %)	51(26.6%)	28(23.1%)	23(32.4%)	0.162
Nosocomial infection (n, %)	74(38.5%)	50(41.3%)	24(33.8%)	0.303
*GPB (n, %)	86 (44.8%)	54 (44.6%)	32 (45.1%)	0.953
MV (n, %)	167(87%)	105(86.8%)	62(87.3%)	0.914
CRRT (n, %)	48(25%)	26(21.5%)	22(31%)	0.143
LOS (days) (median, IQR)	18(5-35.75)	30(17.5-43)	4(2-13)	0.001
PICU stay (days) (median, IQR)	7.5(3-17)	12(5-25)	3(1-7)	<0.001

AKI, acute kidney injury; ALB, albumin; CRP, C-reactiveprotein; CRRT, continuous renal replacement therapy; DIC, disseminated intravascular coagulation; Fib, fibrinogen; GPB, gram-positive bacteria; MV, mechanical ventilation; IQR, inter quartile range; ISTH, international society on thrombosis and haemostasis; Los, Length of stay; PCT, Procalcitonin; P/F value, PaO2/FiO2; PICU, Pediatric intensive care unit; pSOFA, pediatric sequential organ failure assessment; SCr, serum creatinine; SIC, Sepsis-induced coagulopath scores; WBC, White blood cell.

*GPB: Double organism growth was detected in 8 cultures, determine whether the pathogen was Gram-positive or Gram-negative based on the results of the first blood culture.

*Underlying conditions: children with underlying comorbidities, in this study, the underlying diseases include hematologic malignancies, bone marrow transplantation, rheumatic and autoimmune diseases, primary immunodeficiencies, malnutrition, and diabetes mellitus.

The median age of these patients, of whom 114 (59.4%) were male, was 16 (6-84) months and the median weight was 10.25 (7-22.75) kg. Septic shock was present in 100 cases (52.1%), respiratory failure in 158 cases (82.3%), liver function impairment in 108 cases (56.3%), acute kidney injury in 39 cases (20.3%), and underlying conditions were present in 51 cases (26.6%) at presentation.

During the 28-day hospital stay, 71 patients (36.98%) died. The endpoint of the study was the 28-day in-hospital mortality (all-cause). Patients discharged from the hospital within 28 days were deemed alive unless proven otherwise. When comparing the survival and non-survival groups, in terms of laboratory tests, there was no significant difference in CRP. There were also no significant differences in respiratory failure, liver function damage, underlying conditions, gram-positive bacteria and nosocomial infection. Additionally, the requirements of MV and CRRT were similar between the two groups.

The levels of WBC (9.67 *vs*. 5.96 × 10^9/L, P < 0.001), ALB (30.4 *vs*. 26.4 g/l, *P* < 0.001), and P/F value (345.71 vs.147.06, *P <* 0.001) in the survival group were significantly higher than in the no-survival group, while the opposite was true for PCT (6.34 *vs*. 14.54 ng/ml, *P =* 0.039), SCr (28.2 *vs*. 41 mmol/l, *P <* 0.001), pSOFA (8 *vs*. 12, *P <* 0.001), ISTH-DIC (3 *vs*. 5, *P <* 0.001), and SIC (3 *vs*. 4, *P <* 0.001). The prevalence of septic shock (42.1% *vs*. 69%, P < 0.001) and acute kidney injury (14% *vs*. 31%, P = 0.005) in the survival group were significantly lower than in the non-survival group. Compared with the two groups, LOS (30 *vs*. 4 days, *P =* 0.001) and PICU stay (12 *vs*. 3 days, *P <* 0.001) in survival the group were significantly higher than in the no- survival group.

### 3.3 Analyses of risk factors for 28-day in-hospital mortality of BSI

Univariate analysis revealed that ALB, pSOFA, P/F value, Septic shock, and AKI were associated with 28-day in-hospital mortality (**Table 2**). Multivariate analysis further showed that pSOFA (OR 1.176; 95% CI: 1.046-1.321, *p* = 0.007) and P/F value (OR 0.994; 95% CI: 0.991-0.997, *P <* 0.001) were independently correlated with 28-day in-hospital mortality (**Table 2**). Conversely, septic shock and AKI were not identified as independent risk factors for 28-day in-hospital mortality.

**Table 2:** Logistic regression analysis of risk factors for 28-day mortality in 192 children with BSI.

	Univariate analysis			Multivariate analysis		
	OR	95%CI	P value	OR	95%CI	P value
ALB	0.915	0.874-0.959	<0.001	0.949	0.898-1.002	0.061
pSOFA	1.328	1.203-1.467	<0.001	1.176	1.046-1.321	0.007*
P/F value	0.992	0.99-0.995	<0.001	0.994	0.991-0.997	<0.001*
Septic shock	3.057	1.646-5.677	<0.001	1.784	0.839-3.792	0.132
AKI	2.747	1.339-5.633	0.006	1.435	0.564-3.655	0.449

AKI, acute kidney injury; ALB, albumin; BSI, bloodstream infection; P/F value, PaO2/FiO2; pSOFA, pediatric sequential organ failure assessment.

*Indicates statistically significant results, *P* < 0.05.

### 3.4 Microbial findings

**Figure 2:**
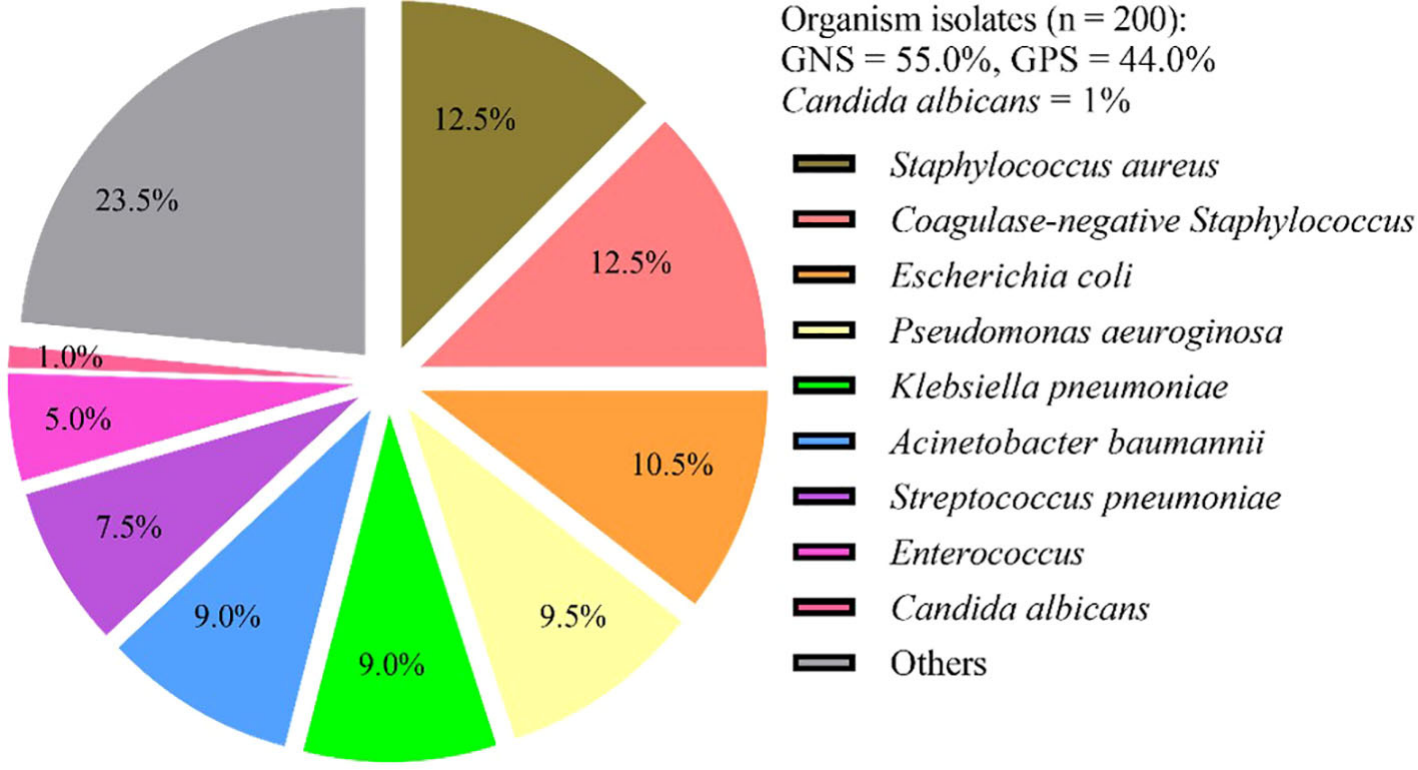
Frequency and distribution of bacteria isolated from patients investigated for BSI in PICU. BSI, Bloodstream infection; GNB, gram-negative bacteria; GPB, gram-positive bacteria; Others included (n = 47): *Salmonella* (n = 6), *Streptococcus paldyne* (n = 5), *Serratia marcescens* (n = 5), *Enterobacter aerogenes* (n = 3), *Haemophilus influenzae* (n = 4), *Streptococcus agalactiae* (n = 3), *Enterobacter cloacae* (n = 3), *Streptococcus pyogenes* (n = 2), *Streptococcus salivarius* (n = 2), *Burkholderia cepacia* (n = 2), *Streptococcus bovis* (n = 1), *Acinetobacter jeonii* (n = 1), *Bacillus pallanus* (n = 1), *Stenotrophomonas maltophila* (n = 1), *Bordetella bronchitis* (n = 1), *Tsukamura genus* (n = 1), *Burkholderia* (n = 1), *Acid Klebber* (n = 1), *Citrobacterium festoni* (n = 1), *Acinetobacter lofe* (n = 1), *Xylose-oxidizing Chromobacterium* (n = 1), *Clostridium septicaemic meningitis* (n = 1).

During the study period, a total of 200 pathogenic bacteria were isolated from blood cultures in PICU. Among these, 110 strains (55.0%) were identified as Gram-negative bacteria, 88 strains (44.0%) were identified as gram-positive bacteria, and 2 strains (1%) were identified as *Candida albicans* (**Table 3**, **Figure 2**). Double organism growth was detected in 8 cultures. The most commonly isolated pathogens were *Staphylococcus aureus*, *Coagulase-negative Staphylococcus*, and *Escherichia coli*. Among Gram-negative bacteria, *Escherichia coli* (21 strains, 10.5%), *Pseudomonas aeuroginosa* (19 strains, 9.5%), *Klebsiella pneumoniae* (18 strains, 9.0%), and *Acinetobacter baumannii* (18 strains, 9.0%) were the most prevalent. Among gram-positive bacteria, *Staphylococcus aureus* (25 strains, 12.5%), *Coagulase-negative Staphylococcus* (25 strains, 12.5%), *Streptococcus pneumoniae* (15 strains, 7.5%), and *Enterococcus* (10 strains, 5.0%) were the most common.

**Table 3:** Microbiology of BSI children admitted in the PICU.

Organism	Subjects n=200	%
Gram-negative bacteria	110	55.0
*E. coli*	21	10.5
*P. aeuroginosa*	19	9.5
*K. pneumoniae*	18	9.0
*A. baumannii*	18	9.0
Gram-positive bacteria	88	44.0
*S. aureus*	25	12.5
*CoNS*	25	12.5
*SP*	15	7.5
*Enterococcus*	10	5.0
Fungi	2	1.0
*Candida albicans*	2	1.0

A. baumannii, Acinetobacter baumannii; CoNS, Coagulase-negative staphylococci; E. coli, Escherichia coli; K. pneumoniae, Klebsiella pneumoniae; P. aeruginosa, Pseudomonas aeuroginosa; S.aureus, Staphylococcus aureus; SP, Streptococcus pneumoniae.

### 3.5 Antimicrobial resistance pattern of main bacterial isolates

As depicted in **Table 4**, *Staphylococcus aureus (S. aureus)* showed a resistance of 95.0%, 23.1%, 4.8% and 65.0% to penicillin, tetracycline, trimethoprim-sulfamethoxazole and erythromycin, respectively. The detection rate of methicillin resistant *Staphylococcus aureus* (MRSA) was 65.0%. *Coagulase-negative staphylococci* (*CoNS*) showed resistance rates ≥ 80% to penicillin and erythromycin. *Streptococcus pneumoniae (SP)* exhibited the highest resistance rates to tetracycline (100%) and erythromycin (91.7%). *Enterococcus* showed 50.0% resistance to penicillin. Vancomycin or linezolid-resistant strains were not isolated in gram-positive bacteria.

**Table 4:** Antimicrobial resistance rates of main gram-positive bacteria isolated from patients investigated for BSI in PICU.

Bacterium	PEN (%)	OXA (%)	VAN (%)	TEC (%)	LZD (%)	TCY (%)	LVX (%)	RIF (%)	SXT (%)	CHL (%)	ERY (%)
*S.aureus* (n=25)	95.0	65.0	0	0	0	23.1	0	0	4.8	0	65.0
*CoNS* (n=25)	91.7	83.3	0	0	0	28.6	41.2	35.7	45.8	NA	81.0
*SP* (n=15)	8.3	NA	0	0	0	100	0	0	58.3	0	91.7
*Enterococcus* (n=10)	50.0	NA	0	0	0	60.0	42.9	NA	50.0	NA	50.0

CHL, chloramphenicol; *CoNS*, *Coagulase-negative staphylococci*; ERY, erythromycin; LVX, levofloxacin; LZD, linezolid; NA, not applicable; OXA, oxacillin; PEN, Penicillin G; R, resistance; RIF, rifampicin; *S.aureus*, *Staphylococcus aureus*; *SP*, *Streptococcus pneumoniae*; SXT, trimethoprim-sulfamethoxazole; TCY, tetracycline; TEC, teicoplanin; VAN, vancomycin.

As shown in **Table 5**, high frequencies of resistance to ampicillin-sulbactam (55.6%), aztreonam (52.9%), ceftriaxone (85.7%), and trimethoprim-sulfamethoxazole (72.2%) were observed among *Escherichia coli (E. coli)*. Minimal resistance frequency of *E. coli* was detected to amikacin (0%), meropenem (17.1%), and piperacillin/tazobactam (16.7%). *Klebsiella pneumoniae (K. pneumoniae)* demonstrated a resistance of 11.1%, 38.5%, 38.5%, and 23.1% to amoxicillin/clavulanate, aztreonam, cefepime, and imipenem, respectively. *Pseudomonas aeuroginosa (P. aeuroginosa)* of the resistance rates was 14.3%, 7.7%, 7.7%, and 15.0% to amikacin, cefepime, levofloxacin, and imipenem, respectively. *Acinetobacter baumannii (A. baumannii)* showed resistance rates exceeding 50% against all tested antibiotics.

**Table 5:** Antimicrobial resistance rates of main gram- negative bacteria isolated from patients investigated for BSI in PICU.

Bacterium	AMK (%)	AMC (%)	SAM (%)	ATM (%)	FEP (%)	CRO (%)	CIP (%)	LEV (%)	GEN (%)	MEM (%)	IPM (%)	TZP (%)	SXT (%)
*E. coli* (n=21)	0	36.4	55.6	52.9	29.4	85.7	47.1	44.4	27.8	17.1	22.2	16.7	72.2
*K. pneumoniae* (n=18)	23.1	11.1	30.8	38.5	38.5	25.0	15.4	8.3	23.1	20.0	23.1	23.1	23.1
*P. aeuroginosa* (n=19)	14.3	100	100	9.1	7.7	100	7.7	7.7	14.3	15.4	15.0	14.3	100
*A. baumannii* (n=18)	62.5	100	90.0	100	72.7	81.8	75.0	58.3	75.0	60.0	66.7	50.0	66.7

*A. baumannii*, *Acinetobacter baumannii*; AMC, amoxicillin/clavulanate; AMK, amikacin; ATM, aztreonam; CIP, ciprofloxacin; CRO, ceftriaxone; *E. coli*, *Escherichia coli*; FEP, cefepime; GEN, gentamicin; IPM, imipenem; *K. pneumoniae*, *Klebsiella pneumoniae*; LEV, levofloxacin; MEM, meropenem; *P. aeruginosa*, *Pseudomonas aeuroginosa*; SAM, ampicillin-sulbactam; SXT, trimethoprim-sulfamethoxazole; TZP, piperacillin/ tazobactam.

### 3.6 The distribution of special antimicrobial resistance phonotypes

**Figure 3:**
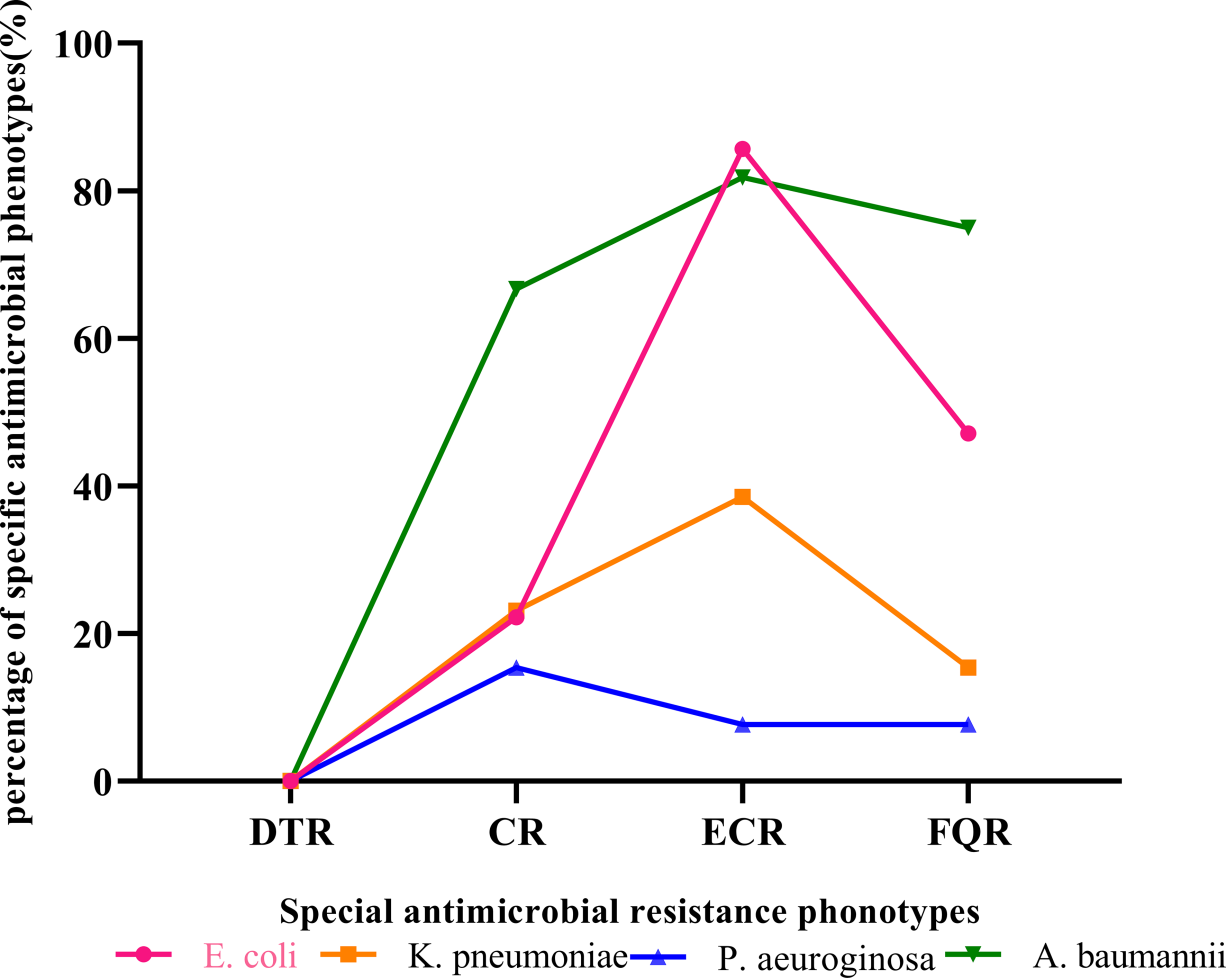
The special antimicrobial resistance phonotypes of main gram- negative bacteria isolated from patients investigated for BSI in PICU. *A. baumannii, Acinetobacter baumannii*; CR, carbapenem resistance; DTR, difficult-to-treat resistance; *E. coli, Escherichia coli*; ECR, extended-spectrum cephalosporin resistance; FQR, fluoroquinolone resistance; *K. pneumoniae, Klebsiella pneumoniae*; *P. aeruginosa, Pseudomonas aeuroginosa*.

As shown in **Table 6** and **Figure 3**, the detection rates of CR, ECR, and FQR were 22.2%, 85.7%, and 47.1% in *E. coli*; 23.1%, 38.5%, and 15.4% in *K. pneumoniae*; 15.4%, 7.7%, and 7.7% in *P. aeruginosa*; and 66.7%, 81.8%, and 75.0% in *A. baumannii*, respectively. DTR strains were not isolated in main gram- negative bacteria. The detection rate of CR in *A baumannii* (66.7%) was higher than that in *P aeruginosa* (15.4%). The detection rates of ECR and FQR in *E. coli* were higher than those in *K. pneumoniae*. *A. baumannii* exhibited the highest levels of CR and FQR, while *E coli* demonstrated the highest level of ECR.

**Table 6:** The special antimicrobial resistance phonotypes of main gram- negative bacteria isolated from patients investigated for BSI in PICU. .

	*E. coli* (%)	*K. pneumoniae* (%)	*P. aeuroginosa* (%)	*A. baumannii* (%)
DTR	0.0	0.0	0.0	0.0
CR	22.2	23.1	15.4	66.7
ECR	85.7	38.5	7.7	81.8
FQR	47.1	15.4	7.7	75.0

*A. baumannii*, *Acinetobacter baumannii*; CR, carbapenem resistance; DTR, difficult-to-treat resistance; *E. coli*, *Escherichia coli*; ECR, extended-spectrum cephalosporin resistance; FQR, fluoroquinolone resistance; *K. pneumoniae*, *Klebsiella pneumoniae*; *P. aeruginosa*, *Pseudomonas aeruginosa*.

## 4 Discussion

In this study, the overall positive rate of blood cultures from 2015 to 2023 was 4.23%, which is consistent with findings from a previous study (Limmathurotsakul, 2017). After excluding contaminants and duplicates, the adjusted average positive rate of blood cultures was 2.21%. Sepsis can be induced by a wide range of pathogens, with bacterial infections accounting for the majority of cases. However, up to 42% of sepsis episodes are culture-negative (Lin et al., 2018), indicating the potential involvement of non-bacterial etiologies (Phua et al., 2013). Additionally, some studies have identified viruses as another significant cause of sepsis, particularly in pediatric populations (Lin et al., 2018), which may help explain the relatively low positive rate of blood cultures observed in our study. we conducted a retrospective analysis of clinical data from a single-center PICU over a 9-year period to investigate BSI-induced severe sepsis. The 28-day in-hospital mortality rate was 36.98%. Among the patients, 26.6% had underlying conditions, 82.3% experienced respiratory failure, 56.3% exhibited liver function impairment, 52.1% developed septic shock, and 20.3% suffered from acute kidney injury. In children with BSI-induced severe sepsis, mortality was higher in those with high pSOFA and low P/F values. The mortality rates for pediatric severe sepsis or septic shock have been reported as high as 40% (Weiss et al., 2015; Wang et al., 2014). A retrospective study analyzed 132 children with BSI-induced severe sepsis, revealing a mortality rate of 28.8% (Dou et al., 2024). Taddei et al. (Allel et al., 2023) recently noted crude mortality rates for ICU-acquired bloodstream infections exceeding 40%. In our study, the 28-day mortality rate was 36.98%. The SOFA score has been recommended by the Society of Critical Care Medicine and the European Society of Intensive Care Medicine for predicting mortality in sepsis. Specifically, a 2-point increase in the SOFA score is associated with a 10% increase in mortality (Shankar-Hari et al., 2016). A systematic review and meta-analysis noted that SOFA showed the highest sensitivity and specificity in predicting in-hospital mortality in sepsis (Qiu et al., 2023). Our multivariate analysis revealed that both pSOFA (OR 1.176; 95% CI: 1.046-1.321, *p* = 0.007) and P/F value (OR 0.994; 95% CI: 0.991-0.997, *P <* 0.001) were independently correlated with 28-day in-hospital mortality.

Organ dysfunction syndrome commonly developed in severe sepsis and was associated with higher morbidity and mortality compared to severe sepsis without organ dysfunction syndrome (Lin et al., 2017). Agyeman et al. (2017) demonstrated a strong association between organ dysfunction and mortality, reporting a notable increase to 17% with at least one organ dysfunction and 29% with two or more organ dysfunctions. In our study, although not statistically significant, the survival group eb3xhibited lower rates of respiratory failure and liver function impairment compared to the non-survival group. Septic shock (42.1% *vs*. 69%, P < 0.001) and acute kidney injury (14% *vs*. 31%, P = 0.005) were significantly less frequent in the survival group than in the non-survival group. These findings collectively indicate higher disease severity in the non-survival group than in the survival group. Hence, continuous assessment of SOFA score, P/F value index, and preservation of organ function may enhance the prognosis of children with BSI-induced severe sepsis.

In this study, a total of 200 pathogenic bacteria were isolated from blood cultures in the PICU. Among these, 110 strains (55.0%) were identified as gram-negative bacteria, 88 strains (44.0%) as gram-positive bacteria, and 2 strains (1%) as *Candida albicans*. The most frequently isolated pathogens were *Staphylococcus aureus (S.aureus)* (12.5%), *Coagulase-negative Staphylococcus* (*CoNS*) (12.5%), and *Escherichia coli* (*E. coli*) (10.5%), consistent with findings from previous studies (Kontula et al., 2021; Dou et al., 2024; Fu et al., 2021). According to the Infectious Disease Surveillance of Pediatrics (ISPED) program in China from 2016 to 2020, a total of 288,377 clinical strains were isolated from 11 member units, with Gram-positive and Gram-negative bacteria accounting for 42.1% and 57.9%, respectively. Among these, the top two pathogenic bacteria derived from blood specimens were *CoNS* (46.3%), and *E coli* (6.0%) (Fu et al., 2021). A study conducted in PICUs in China on bloodstream infection pathogens reported 31.8% gram-positive bacteria, 65.9% gram-negative bacteria, and 2.27% fungi (Dou et al., 2024). In a population-based study of bloodstream infection, Kontula et al. (Kontula et al., 2021) showed among all BSIs, gram-positive bacteria caused 46% of infections, gram-negative bacteria 46%, fungi 1.5%. *E. coli* was the most common causative pathogen (29%), followed by *S. aureus* (13%), *CoNS* (8%).

There has been an increase in bacteremia caused by *S aureus*, *CoNS* and gram-negative pathogens across all age groups in children (Pai et al., 2015). Several surveys had demonstrated a rising trend in multidrug-resistant bloodstream infections (MDR BSIs) over time (Kontula et al., 2021; de Kraker et al., 2013). In our study, we analyzed the antimicrobial resistance pattern of the main bacterial isolates. In resistance patterns, all the main gram-positive bacteria were susceptible to vancomycin and linezolid, similar to the study of ZY L et al (Lyu et al., 2023). *S. aureus* exhibited frequent resistance to Penicillin and erythromycin, while showing high susceptibility to glycopeptides. Some studies have noted inferior outcomes were reported in methicillin-susceptible *S aureus* bacteremia (MSSA-B) treated with glycopeptides compared with β-lactams (McMullan et al., 2020; 2016; McDanel et al., 2015). In clinical practice, glycopeptides are preferred for methicillin-resistant S aureus bacteremia (MRSA-B), whereas for MSSA-B, β-lactams should be preferred over glycopeptides.

Infections caused by resistant Gram-negative bacteria are increasingly concerning in both developing and developed countries (Pai et al., 2015). In present study, *E. coli* showed high resistance to aztreonam, ceftriaxone, cefuroxime and trimethoprim-sulfamethoxazole. Minimal resistance was observed in *E. coli* against amikacin, piperacillin/tazobactam, and meropenem, consistent with findings from other studies (Dou et al., 2024; Legese et al., 2022). We observed an alarming resistance of *K. pneumoniae* to meropenem (20.0%), even higher than reported in another study (Legese et al., 2022). *A baumannii* exhibited resistance rates exceeding 50% against all tested drugs, notably high resistance to meropenem (60.0%), consistent with previous studies (Dou et al., 2024; Legese et al., 2022).

The pathogens of bloodstream infection vary across regions, and multidrug resistance (MDR) poses a threat to public health. Our study showed that the validity rate of the carbapenem-resistant *E. coli* (CR-ECO) was 22.2%, which was lower than that of the carbapenem-resistant *K. pneumoniae* (CR-KPN) (23.1%). Compared to the study by ZY L et al (Lyu et al., 2023), our detection rates of CRECO were higher, while the detection rates of CR-KPN were significantly lower. In our study, among the non-fermentative gram-negative bacilli isolates, 15.4% and 15% of the *P. aeruginosa*, were resistant to meropenem and imipenem. Furthermore, the resistance rates of *A. baumannii* to meropenem and imipenem were 60.0% and 66.7%. The detection rates of ECR and FQR in *E. coli* were higher than those in *K. pneumoniae*. *A. baumannii* exhibited the highest levels of CR and FQR, while *E coli* demonstrated the highest level of ECR. In the study of ZY L et al (Lyu et al., 2023), *K. pneumoniae* demonstrated the highest level of ECR, *E. coli* the highest level of FQR, and *A. baumannii* the highest level of CR. There were significant differences in medication habits and drug resistance among different regions. Monitoring local multidrug resistance (MDR) and selecting antibiotics reasonably based on susceptibility testing were crucial.

## 5 Conclusion

In conclusion, among children with BSI-induced severe sepsis, higher mortality was observed in those with elevated pSOFA scores and lower P/F values. The common pathogen of BSI−induced severe sepsis in PICU is Gram−negative bacteria. The three most frequently isolated pathogens were *Staphylococcus aureus, Coagulase-negative Staphylococcus, and Escherichia coli*. The pathogens of bloodstream infection vary across regions, monitoring local multidrug resistance (MDR) and selecting antibiotics reasonably based on susceptibility testing were crucial.

## 6 Limitation

This study has several limitations. Firstly, being retrospective, it was confined to cases and data from a single center. Consequently, these findings may not generalize to centers with different patient populations or antibiotic profiles. Secondly, the study focused exclusively on patients with documented bloodstream infections, potentially introducing selection bias since not all bacteremia cases were confirmed by positive blood cultures. Thirdly, retrospective studies could not further explore the mechanism of MDR resistance, and we did not analyze the molecular mechanisms of the drug-resistant strains.

## Data Availability

The original contributions presented in the study are included in the article/supplementary material. Further inquiries can be directed to the corresponding authors.

## Glossary

*A. baumannii*: *Acinetobacter baumannii*
AKI: acute kidney injury
ALB: albumin
AMK: amikacin
AMC: amoxicillin/clavulanate
ATM: aztreonam
BSI: bloodstream infection
CHL: chloramphenicol
CIP: ciprofloxacin
*CoNS*: *Coagulase-negative staphylococci*
CR-ECO: carbapenem-resistant *E. coli*
CR-KPN: carbapenem-resistant *K. pneumoniae*
CRO: ceftriaxone
CRP: C-reactive protein
CRRT: continuous renal replacement therapy
CXM: cefuroxime
*E. coli*: *Escherichia coli*
DTR: difficult-to-treat resistance
ECR: extended-spectrum cephalosporin resistance
ERY: erythromycin
FEP: cefepime
Fib: fibrinogen
FOX: cefoxitin
FQR: fluoroquinolone resistance
GEN: gentamicin
GNB: gram-negative bacteria
GPB: gram-positive bacteria
I: intermediate
IQR: inter quartile range
ISTH-DIC: international society on thrombosis and hemostasis
*K. pneumoniae*: *Klebsiella pneumoniae*
Los: Length of stay
P/F value: oxygenation index (PaO2/FiO2)
LVX: levofloxacin
MEM: meropenem
MV: mechanical ventilation
NA: not applicable
OXA: oxacillin
*P. aeruginosa*: *Pseudomonas aeuroginosa*
PCT: Procalcitonin
PEN: Penicillin
PICU: Pediatric intensive care unit
pSOFA: pediatric sequential organ failure assessment
R: resistance
RIF: rifampicin
*S.aureus*: *Staphylococcus aureus*
SAM: ampicillin-sulbactam
SCr: serum creatinine
SIC: Sepsis-induced coagulopath scores
*SP*: *Streptococcus pneumoniae*
SXT: trimethoprim-sulfamethoxazole
TCY: tetracycline
TZP: piperacillin/tazobactam
VAN: vancomycin
IPM: imipenem
WBC: White blood cell

## References

[B1] Agyeman P. K. A. Schlapbach L. J. Giannoni E. Stocker M. Posfay-Barbe K. M. Heininger U.. (2017). Epidemiology of blood culture-proven bacterial sepsis in children in Switzerland: a population-based cohort study. Lancet Child Adolesc. Health 1, 124–133. doi: 10.1016/s2352-4642(17)30010-x 30169202

[B2] Allel K. Stone J. Undurraga E. A. Day L. Moore C. E. Lin L.. (2023). The impact of inpatient bloodstream infections caused by antibiotic-resistant bacteria in low- and middle-income countries: A systematic review and meta-analysis. PloS Med. 20, e1004199. doi: 10.1371/journal.pmed.1004199 37347726 PMC10287017

[B3] de Kraker M. E. Jarlier V. Monen J. C. Heuer O. E. van de Sande N. Grundmann H. (2013). The changing epidemiology of bacteraemias in Europe: trends from the European Antimicrobial Resistance Surveillance System. Clin. Microbiol. Infect. 19, 860–868. doi: 10.1111/1469-0691.12028 23039210

[B4] Dou J. Y. Zhou Y. P. Cui Y. Sun T. Shi J. Y. Xiong X.. (2024). Pathogenic characteristics and influence factors of bloodstream infection-induced severe sepsis in pediatric intensive care unit. Zhonghua. Yi. Xue. Za. Zhi. 104, 198–204. doi: 10.3760/cma.j.cn112137-20230729-00115 38220445

[B5] Fu P. Xu H. Jing C. Deng J. Wang H. Hua C.. (2021). Bacterial epidemiology and antimicrobial resistance profiles in children reported by the ISPED program in China 2016 to 2020. Microbiol. Spectr. 9, e0028321. doi: 10.1128/Spectrum.00283-21 34730410 PMC8567242

[B6] Goldstein B. Giroir B. Randolph A. (2005). International pediatric sepsis consensus conference: definitions for sepsis and organ dysfunction in pediatrics. Pediatr. Crit. Care Med. 6, 2–8. doi: 10.1097/01.Pcc.0000149131.72248.E6 15636651

[B7] Goudie A. Dynan L. Brady P. W. Rettiganti M. (2014). Attributable cost and length of stay for central line-associated bloodstream infections. Pediatrics 133, e1525–e1532. doi: 10.1542/peds.2013-3795 24799537 PMC4258643

[B8] Kontula K. S. K. Skogberg K. Ollgren J. Järvinen A. Lyytikäinen O. (2021). Population-based study of bloodstream infection incidence and mortality rates, Finland 2004-2018. Emerg. Infect. Dis. 27, 2560–2569. doi: 10.3201/eid2710.204826 34546161 PMC8462341

[B9] Laupland K. B. Leal J. R. (2020). Defining microbial invasion of the bloodstream: a structured review. Infect. Dis. (Lond). 52, 391–395. doi: 10.1080/23744235.2020.1727948 32064990

[B10] Legese M. H. Asrat D. Swedberg G. Hasan B. Mekasha A. Getahun T.. (2022). Sepsis: emerging pathogens and antimicrobial resistance in Ethiopian referral hospitals. Antimicrobial. Resistance. Infect. Control. 11, 83. doi: 10.1186/s13756-022-01122-x PMC9195281 35698179

[B11] Limmathurotsakul D. (2017). Causes and outcomes of sepsis in southeast Asia: a multinational multicentre cross-sectional study. Lancet Global Health 5, e157–e167. doi: 10.1016/s2214-109x(17)30007-4 28104185 PMC5332551

[B12] Lin G. L. McGinley J. P. Drysdale S. B. Pollard A. J. (2018). Epidemiology and immune pathogenesis of viral sepsis. Front. Immunol. 9. doi: 10.3389/fimmu.2018.02147 PMC6170629 30319615

[B13] Lin J. C. Spinella P. C. Fitzgerald J. C. Tucci M. Bush J. L. Nadkarni V. M.. (2017). New or progressive multiple organ dysfunction syndrome in pediatric severe sepsis: A sepsis phenotype with higher morbidity and mortality. Pediatr. Crit. Care Med. 18, 8–16. doi: 10.1097/pcc.0000000000000978 28060151 PMC7261134

[B14] Lyu Z. Y. Zhen J. H. Meng Q. Y. Zhou W. An J. Y. Dong F. (2023). Bacterial etiology and antimicrobial resistance pattern of pediatric bloodstream infections in Beijing 2015-2019. Infect. Drug Resistance. 16, 6297–6308. doi: 10.2147/idr.S426000 PMC10540788 37780532

[B15] Martinón-Torres F. Salas A. Rivero-Calle I. Cebey-López M. Pardo-Seco J. Herberg J. A.. (2018). Life-threatening infections in children in Europe (the EUCLIDS Project): a prospective cohort study. Lancet Child Adolesc. Health 2, 404–414. doi: 10.1016/s2352-4642(18)30113-5 30169282

[B16] McDanel J. S. Perencevich E. N. Diekema D. J. Herwaldt L. A. Smith T. C. Chrischilles E. A.. (2015). Comparative effectiveness of beta-lactams versus vancomycin for treatment of methicillin-susceptible Staphylococcus aureus bloodstream infections among 122 hospitals. Clin. Infect. Dis. 61, 361–367. doi: 10.1093/cid/civ308 25900170

[B17] McMullan B. J. Bowen A. Blyth C. C. Van Hal S. Korman T. M. Buttery J.. (2016). Epidemiology and mortality of staphylococcus aureus bacteremia in Australian and New Zealand children. JAMA Pediatr. 170, 979–986. doi: 10.1001/jamapediatrics.2016.1477 27533601

[B18] McMullan B. J. Campbell A. J. Blyth C. C. McNeil J. C. Montgomery C. P. Tong S. Y. C.. (2020). Clinical management of staphylococcus aureus bacteremia in neonates, children, and adolescents. Pediatrics 146, e20200134. doi: 10.1542/peds.2020-0134 32759380

[B19] Mohamed El-Mashad G. Said El-Mekkawy M. Helmy Zayan M. (2020). Paediatric sequential organ failure assessment (pSOFA) score: A new mortality prediction score in the paediatric intensive care unit. Pediatr. (Engl. Ed). 92, 277–285. doi: 10.1016/j.anpedi.2019.05.018 31784324

[B20] Munro C. Zilberberg M. D. Shorr A. F. (2024). Bloodstream infection in the intensive care unit: evolving epidemiology and microbiology. Antibiot. (Basel. Switzerland). 13, 123. doi: 10.3390/antibiotics13020123 PMC10886070 38391509

[B21] Pai S. Enoch D. A. Aliyu S. H. (2015). Bacteremia in children: epidemiology, clinical diagnosis and antibiotic treatment. Expert Rev. Anti-infect. Ther. 13, 1073–1088. doi: 10.1586/14787210.2015.1063418 26143645

[B22] Phua J. Ngerng W. See K. Tay C. Kiong T. Lim H.. (2013). Characteristics and outcomes of culture-negative versus culture-positive severe sepsis. Crit. Care (London. England). 17, R202. doi: 10.1186/cc12896 PMC4057416 24028771

[B23] Qiu X. YP L. Zhou R. X. (2023). SIRS, SOFA, qSOFA, and NEWS in the diagnosis of sepsis and prediction of adverse outcomes: a systematic review and meta-analysis. Expert Rev. Anti-infect. Ther. 21, 891–900. doi: 10.1080/14787210.2023.2237192 37450490

[B24] Rhee C. Jones T. M. Hamad Y. Pande A. Varon J. O’Brien C.. (2019). Prevalence, underlying causes, and preventability of sepsis-associated mortality in US acute care hospitals. JAMA Netw. Open 2, e187571. doi: 10.1001/jamanetworkopen.2018.7571 30768188 PMC6484603

[B25] Rudd K. E. Johnson S. C. Agesa K. M. Shackelford K. A. Tsoi D. Kievlan D. R.. (2020). Global, regional, and national sepsis incidence and mortality 1990-2017: analysis for the global burden of disease study. Lancet (London. England). 395, 200–211. doi: 10.1016/s0140-6736(19)32989-7 31954465 PMC6970225

[B26] Shankar-Hari M. Phillips G. S. Levy M. L. Seymour C. W. Liu V. X. Deutschman C. S.. (2016). Developing a new definition and assessing new clinical criteria for septic shock: for the third international consensus definitions for sepsis and septic shock (Sepsis-3). Jama 315, 775–787. doi: 10.1001/jama.2016.0289 26903336 PMC4910392

[B27] Singer M. Deutschman C. S. Seymour C. W. Shankar-Hari M. Annane D. Bauer M.. (2016). The third international consensus definitions for sepsis and septic shock (Sepsis-3). JAMA 315, 801–810. doi: 10.1001/jama.2016.0287 26903338 PMC4968574

[B28] Tabah A. Lipman J. Barbier F. Buetti N. Timsit J. F. On Behalf Of The Escmid Study Group For Infections In Critically Ill Patients-Esgcip (2022). Use of antimicrobials for bloodstream infections in the intensive care unit, a clinically oriented review. Antibiot. (Basel. Switzerland). 11, 362. doi: 10.3390/antibiotics11030362 PMC8944491 35326825

[B29] Timsit J. F. Ruppé E. Barbier F. Tabah A. Bassetti M. (2020). Bloodstream infections in critically ill patients: an expert statement. Intensive Care Med. 46, 266–284. doi: 10.1007/s00134-020-05950-6 32047941 PMC7223992

[B30] Wang Y. Sun B. Yue H. Lin X. Li B. Yang X.. (2014). An epidemiologic survey of pediatric sepsis in regional hospitals in China. Pediatr. Crit. Care Med. 15, 814–820. doi: 10.1097/pcc.0000000000000247 25226498

[B31] Weiss S. L. Fitzgerald J. C. Pappachan J. Wheeler D. Jaramillo-Bustamante J. C. Salloo A.. (2015). Global epidemiology of pediatric severe sepsis: the sepsis prevalence, outcomes, and therapies study. Am. J. Respir. Crit. Care Med. 191, 1147–1157. doi: 10.1164/rccm.201412-2323OC 25734408 PMC4451622

[B32] Weiss S. L. Peters M. J. Alhazzani W. Agus M. S. D. Flori H. R. Inwald D. P.. (2020). Surviving sepsis campaign international guidelines for the management of septic shock and sepsis-associated organ dysfunction in children. Intensive Care Med. 46, 10–67. doi: 10.1007/s00134-019-05878-6 32030529 PMC7095013

[B33] World Health Organization (2020). Global report on the epidemiology and burden of sepsis: Current evidence, identifying gaps and future directions. Available online at: https://apps.who.int/iris/bitstream/handle/10665/334216/9789240010789-eng.pdf?ua=1 (Accessed June 8, 2024).

[B34] Zingg W. Hopkins S. Gayet-Ageron A. Holmes A. Sharland M. Suetens C. (2017). Health-care-associated infections in neonates, children, and adolescents: an analysis of paediatric data from the European Centre for Disease Prevention and Control point-prevalence survey. Lancet Infect. Dis. 17, 381–389. doi: 10.1016/s1473-3099(16)30517-5 28089444

